# Molecular Analysis of *Aggregatibacter actinomycetemcomitans* ApiA, a Multi-Functional Protein

**DOI:** 10.3390/pathogens13111011

**Published:** 2024-11-18

**Authors:** Sera Jacob, Luciana Gusmao, Dipti Godboley, Senthil Kumar Velusamy, Nisha George, Helen Schreiner, Carla Cugini, Daniel H. Fine

**Affiliations:** Department of Oral Biology, Rutgers School of Dental Medicine, 110 Bergen, Newark, NJ 07103, USA; serageo14@gmail.com (S.J.); lgusmao@amnh.org (L.G.); godboldv@sdm.rutgers.edu (D.G.); senthil.velusamy@fujifilm.com (S.K.V.); nishageorge.george@gmail.com (N.G.); hschrein@sdm.rutgers.edu (H.S.)

**Keywords:** complement resistance, *Aggregatibacter actinomycetemcomitans*, factor H, epithelial cell binding, auto-aggregation, global regulation

## Abstract

*Aggregatibacter actinomycetemcomitans* ApiA is a trimeric autotransporter outer membrane protein (Omp) that participates in multiple functions, enabling *A. actinomycetemcomitans* to adapt to a variety of environments. The goal of this study is to identify regions in the *apiA* gene responsible for three of these functions: auto-aggregation, buccal epithelial cell binding, and complement resistance. Initially, *apiA* was expressed in *Escherichia coli*. Finally, wild-type *A. actinomycetemcomitans* and an *apiA-*deleted version were tested for their expression in the presence and absence of serum and genes related to stress adaptation, such as oxygen regulation, catalase activity, and Omp proteins. Sequential deletions in specific regions in the *apiA* gene as expressed in *E. coli* were examined for membrane proteins, which were confirmed by microscopy. The functional activity of epithelial cell binding, auto-aggregation, and complement resistance were then assessed, and regions in the *apiA* gene responsible for these functions were identified. A region spanning amino acids 186–217, when deleted, abrogated complement resistance and Factor H (FH) binding, while a region spanning amino acids 28–33 was related to epithelial cell binding. A 13-amino-acid peptide responsible for FH binding was shown to promote serum resistance. An *apiA* deletion in a clinical isolate (IDH781) was created and tested in the presence and/or absence of active and inactive serum and genes deemed responsible for prominent functional activity related to *A. actinomycetemcomitans* survival using qRT-PCR. These experiments suggested that *apiA* expression in IDH781 is involved in global regulatory mechanisms that are serum-dependent and show complement resistance. This is the first study to identify specific *apiA* regions in *A. actinomycetemcomitans* responsible for FH binding, complement resistance, and other stress-related functions. Moreover, the role of *apiA* in overall gene regulation was observed.

## 1. Introduction

In a complex biofilm, the interaction of individual microorganisms with other members of the diverse biofilm microbiome is multifaceted [1]. This is especially true when these complex associations are compounded by the effects of host factors [2]. In a microbiome at homeostasis, there are delicate interspecies interactions driven by the maintenance of intricate physical and metabolic associations that control host innate immune defenses, aimed at detoxifying the assaulting microbiome [3]. Periodontal disease is characterized by the formation of a dysbiotic biofilm and an outgrowth of key pathobionts, which can lead to host tissue destruction and, ultimately, periodontal pockets and bone loss [4]. *Aggregatibacter actinomycetemcomitans*, a well-studied member of the microbiome involved in Stage III Grade C periodontitis (previously called localized aggressive periodontitis; LAgP) which occurs predominantly in adolescents of African descent is of particular interest in dysbiotic associations [5]. *A. actinomycetemcomitans* is a Gram-negative, capnophilic, facultative anaerobic member of the oral microbiota and a critical agent associated with the initial mucosal infection in periodontitis [6]. *A. actinomycetemcomitans* is unique because it has the ability to adapt to diverse environments for its own survival and for the protection of other less adaptable cohorts, especially in the subgingival environment [7]. Our group has studied several of A. *actinomycetemcomitans*’s virulence factors, including the widespread colonization island (WCI), which includes Flp [8], leukotoxin [9], cytolethal distending toxin [10], DispB [11,12], PNAG [13], Aae [14], and ApiA [15]. Of these A. *actinomycetemcomitans* virulence factors, ApiA and its relationship to complement resistance is perhaps the least understood, although recently more information has become available [16,17].

It is proposed that *A. actinomycetemcomitans* initially colonizes the oral mucosa by means of Aae, a monomeric autotransporter adhesin that binds at low concentrations to epithelial receptors in the supragingival domain [15]. Subsequently, when *A. actinomycetemcomitans* reaches higher levels numerically in the supragingival domain, ApiA, a trimeric autotransporter outer membrane protein (Omp), plays a major role in oral colonization, but this accumulation occurs in a linear fashion independent of receptor/adhesin interactions, suggesting auto-aggregation [15]. The successful migration and colonization of *A. actinomycetemcomitans* into the subgingival space occur as a result of *dspB*, an enzyme that disrupts the biofilm’s protective shield and ejects cells from the inner core of the biofilm mass huge distances away from the original biofilm deposition [18]. *A. actinomycetemcomitans*’s subgingival migration is instrumental in the development of a consortia of bacteria that can lead to the destruction of periodontal tissue and the development of periodontitis in susceptible individuals [19,20,21,22]. *A. actinomycetemcomitans*’s subgingival presence provokes an outpouring of gingival crevicular fluid, a serum exudate, which increases the inflammatory burden, resulting in additional colonization and proliferation at the site (5). The initial host response relies on serum-derived complements and host-derived cell-related toxins such as leukotoxin [23].

Many commensals and pathobionts have developed elegant strategies to subvert the cytotoxic effects of host defense systems, allowing for their persistence within this specific subgingival niche [24,25,26,27]. Several oral microbes display serum resistance and blunt the effects of the complement cascade, which can span the classical, lectin, and alternative pathways [24,28,29]. As compared to complement resistance in other pathobionts such as *Porphyromonas gingivalis,* studies of *A. actinomycetemcomitans* have fallen short, perhaps because ApiA, as a trimeric protein in nature, presents major structural challenges [16]. The alternative complement system, which is proposed to be triggered by *A. actinomycetemcomitans* ApiA, involves an enzymatic cascade that ultimately can activate a set of pore-forming proteins, leading to bacterial cell lysis [30]. The alternative pathway is governed by abundant levels of C3 protein, which undergoes a constant low level of spontaneous hydrolysis to C3a (anaphylatoxin) and C3b. In the absence of an antibody-guided response, C3b binds to cell wall components and lipopolysaccharides of “invading” cells [31]. A major regulator of the alternative complement pathway known to bind to and stabilize C3b is Factor H (FH). Factor H prevents convertase from activating the rest of the complement pathway [32,33].

A hallmark of *A. actinomycetemcomitans* biology is its early resistance to the effects of serum by the disruption of the complement cascade [24,34]. ApiA, a 295-amino-acid outer membrane protein, is conserved in *A. actinomycetemcomitans* species, with 99–100% of its nucleotide sequence identity and 100% of its amino acid identity shared among sequenced strains (accession number AB064943). Further *A. actinomycetemcomitans* is characterized by the interaction of ApiA (Omp100) with human Factor H [15,16,35], and since ApiA is randomly localized on the cell surface, it can facilitate binding to FH, which confers serum resistance [29,36,37]. In the absence of ApiA, *A. actinomycetemcomitans* has a reduced ability to survive the effects of serum, indicating a major role for *A. actinomycetemcomitans* ApiA in serum resistance [29,38,39]. There is precedent among other oral microbes, such as *Porphyromonas gingivalis* and *Treponema denticola,* to interact with proteins of the complement pathway [25,40,41,42,43,44].

The overriding goal of the first portion of this study was to determine regions in ApiA responsible for auto-aggregation, epithelial cell binding, and complement resistance. The premise of this study was that a deletion in an *apiA* gene region that failed to show a specific function, such as epithelial cell binding, was proposed to be responsible for that function. All findings were measured in a quantitative manner and were compared to the complete *apiA* gene expressed in *Esherichia coli* (the positive control) or to *E. coli* with an empty plasmid (the negative control). Following this logic, the deleted gene region that failed to show any complement resistance was proposed to be responsible for complement resistance via the alternative pathway. It has been suggested that *A. actinomycetemcomitans* also activates the classical pathway of complement resistance by means of *ompA-1* [38,45].

Outer membrane protein 100, a 100 kDa protein, later termed ApiA, was first identified by Komatsuzawa et al. in 2002 as one of six outer membrane proteins [36,46]. Since that time, it has been shown that ApiA is a multifunctional outer membrane protein that is involved in epithelial cell binding, auto-aggregation, and complement resistance [15]. This study was intentionally limited to surface-related proteins, and as such, our first aim was to visualize surface expression by means of fluorescence and immunogold labeled transmission electron microscopy. This was followed by studies of auto-aggregation and buccal epithelial cell binding to confirm the surface expression. Gene deletions in *apiA* were used to identify supplemental gene regions related to these supplemental functions. Finally, peptides, designed based on gene sequences deemed responsible for functionality, were used to confirm a region thought to be responsible for Factor H binding and complement resistance. This is the first research study to identify specific regions within ApiA potentially responsible for serum resistance in *A. actinomycetemcomitans,* which could be important in the modulation of immune responsiveness and early disease abatement. Further, the exploration of the *apiA* region in *A. actinomycetemcomitans*, IDH781, allowed us to examine the possibility that *apiA* could be involved in the global regulation of other genes critical for *A. actinomycetemcomitans* survival. These findings point to the potential influence of ApiA in *A. actinomycetemcomitans* adaptability in the face of environmental stressors.

## 2. Materials and Methods

### 2.1. Bacterial Strains and Growth Conditions

Bacterial strains used in this study are listed in Table 1. *A. actinomycetemcomitans* IDH781 and IDH781 *apiA* were routinely grown on brain heart infusion (BHI; Becton, Dickinson and company, Franklin Lakes, NJ, USA) agar and/or trypticase soy agar (TSA) supplemented with 0.6% yeast extract (Beckton Dickinson, Franklin Lakes, NJ, USA), 0.8% dextrose, and 0.4% sodium bicarbonate. For liquid cultures, *A. actinomycetemcomitans* was inoculated in BHI broth (Becton, Dickinson and company, Franklin Lakes, NJ, USA) or trypticase soy broth with 0.6% yeast extract (Becton, Dickinson and company, Franklin Lakes, NJ, USA), 0.8% dextrose, and 0.4% sodium bicarbonate. The strains were incubated at 37 °C in a 10% CO_2_ incubator for 16–48 h or in an anaerobic chamber (10% CO_2_, 10% H_2_, and 80% N_2_).

*E. coli* strains were revived from frozen stocks on Luria–Bertani (LB) plates supplemented with kanamycin (30 µg/mL) and incubated overnight at 37 °C. For expression of ApiA and variants in *E. coli*, each strain was inoculated into LB broth containing dextrose (0.5%) and kanamycin (30 µg/mL) and incubated overnight at 37 °C with shaking (220 rpm). After 16 h, the optical density at 600 nm (OD_600_) of the overnight culture was measured. The strains were subcultured in LB broth supplemented with kanamycin (30 µg/mL) to an OD_600_ of 0.05. Once the culture reached an OD_600_ of 0.5, all strains were induced by adding isopropyl β-D-1-thiogalactopyranoside (IPTG; 0.1 mM). The culture was incubated for 45 min to allow for induction. Bacterial cells were pelleted by centrifugation (4000 rpm; 10 min) and washed three times with PBS (4000 rpm; 5 min). Bacterial cell pellets were then resuspended in 3 mL of PBS.

### 2.2. Cloning of Full-Length apiA and Variants into pET-29(b)+

**Figure 1:**
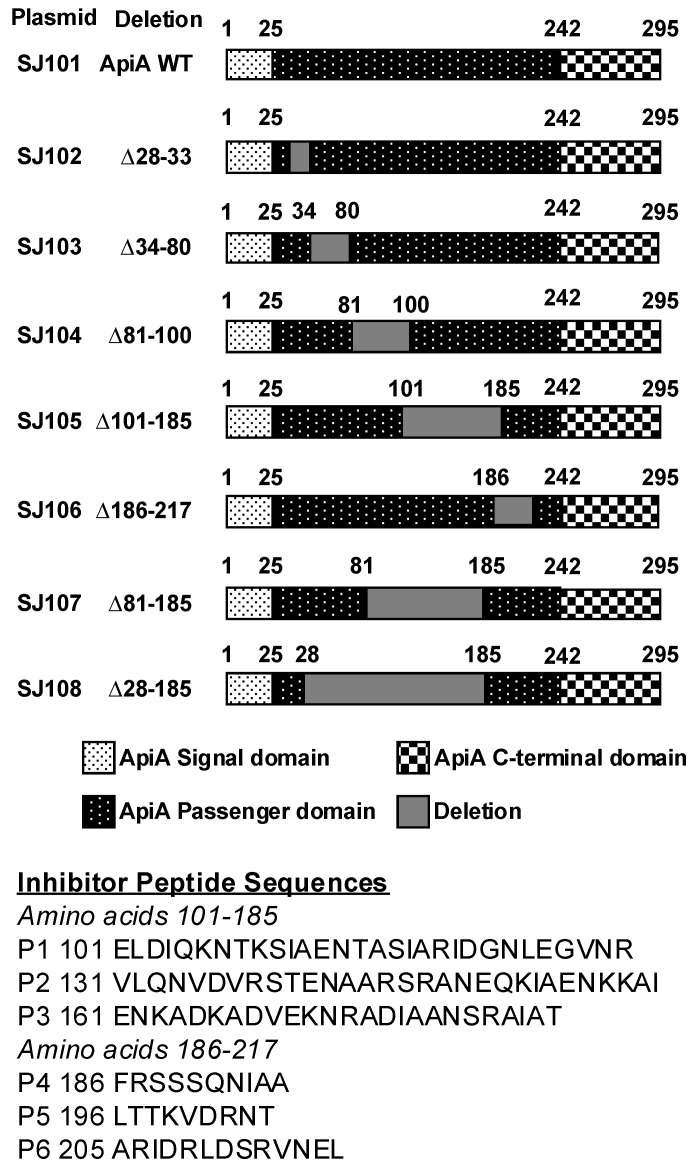
Schematic of the various ApiA sequential deletions used in the functional assays.

The pET-29(b)+ plasmid was purified from *E. coli* using the Qiagen Mini-Prep Kit (Germantown, MD, USA) as per the manufacturer’s recommendation. The plasmid was subjected to double restriction digestion using NdeI and EcoRI high-fidelity restriction enzymes (New England Biolabs (NEB), Ipswich, MA, USA). Primers were designed to PCR amplify the *apiA* gene from IDH781 chromosomal DNA as the template and to include NdeI and EcoRI cleavage sites (Table 2). The desired fragment was amplified using PCR, after which the insert was digested by double digestion using NdeI and EcoRI. The digested inserts were ligated into the digested plasmid using the Instant Sticky-End Ligase Master Mix (New England Biolabs, Ipswich, MA, USA) to create the final ApiA clone. The ligation mixture was used to transform into commercially available chemically competent NEB 5a competent *E. coli* (non-expression host) (Table 1; New England Biolabs, Ipswich, MA, USA) following the manufacturer’s recommendations. The inserted sequence was verified with commercially available T7 promoter and T7 terminator primers (Psomagen, Brooklyn, NY, USA, formerly Macrogen). The recombinant plasmid was transformed into commercially available chemically competent *E. coli* BL21 (DE3, expression host) (Table 1). The plasmid containing the full-length ApiA was designated SJ101 (Figure 1 and Table 1 [35]).

To clone ApiA passenger domain variants, primers were designed to create the desired fragments using overlap extension PCR (Table 2; [48]). The mutants were created by an overlap extension PCR and cut by double restriction digestion using NdeI and EcoRI (Ipswich, MA, USA). The digested inserts were ligated into the digested plasmid using the Instant Sticky-End Ligase Master Mix (NEB, Ipswich, MA, USA) to create the recombinant plasmid. Further, these recombinant plasmids were transformed into commercially available chemically competent NEB 5-alpha-competent *E. coli* and *E. coli* BL21(DE3) (New England Biolabs) following the manufacturer’s recommendations. PCR was performed on single colonies to confirm the insert contained the correct deletion. Plasmids were isolated for sequencing using the same growth conditions and plasmid preparation as previously described to verify the correct constructs. The verified constructs were transformed into commercially available chemically competent *E. coli* BL21(DE3) (New England Biolabs) following the manufacturer’s recommendations for protein expression.

### 2.3. Construction of ApiA Deletion Strain in A. actinomycetemcomitans IDH781

A scarless, markerless deletion approach was used to construct an *apiA* isogenic mutant in strain IDH781 using plasmid pJT1 as previously described [48]. Primers were designed for the PCR amplification of the 1000 bp upstream (apiA NotI UF and apiA UR) and downstream (apiA DF and apiA XhoIR) fragments of *apiA*, which had 15 bp tails complementary to one another to enable fusion between the fragments (Accession: AB064943/IDH781) [47,48]. IDH781 genomic DNA served as the template (~5 ng). NotI and XhoI restriction sites were included in 5′ and 3′ ends of the flanking fragments, respectively, by PCR to enable infusion cloning (Takara Inc., Kusatsu, Japan) into pJT1. An overlap extension PCR (OEPCR) was performed as described previously with equimolar concentrations of the upstream and downstream flanking fragments without end primers [48,49]. The end primers were added, and the second step of PCR using 5 µL template from the first step of PCR was carried out to amplify the fusion product. The PCR fragment was ligated into NotI-XhoI double-digested pJT1 plasmid using an in-fusion cloning strategy (Takara Bio USA, Inc., San Jose, CA, USA). Colonies were screened by PCR for the insert and confirmed by sequencing. The resultant *apiA* deletion plasmid was designated as pSJ13 (Table 1).

Electroporation of pSJ13 into IDH781 was carried out as previously described [15,35,49]. IDH781 was grown for 24 h on BHI plates at 37 °C with supplements in a 10% CO_2_ incubator. Cells were collected with a sterile cotton applicator and suspended in 20 mL ice-cold electroporation buffer (EB; 300 mM sucrose in 2.43 mM phosphate buffer; pH 7.2). These cells were washed three times with EB and centrifuged (8000 rpm) for 10 min at 4 °C. Cells were resuspended using 1/10 volume of EB. To make a homogenous suspension, a hand-held motorized pestle was used to disrupt the clumps. The OD_600_ was adjusted to 0.5–0.6. Forty microliters of washed IDH781 cells were incubated with ~500 ng of pSJ13 plasmid DNA on ice for 5 min and transferred to a 0.2 cm cuvette. The mixture was electroporated with 2.2 kV, 200 Ω, and 25 µF (Gene Pulsar; Bio-Rad, Hercules, CA, USA) and recovered in 1 mL of warm BHI media at 37 °C in a 10% CO_2_ atmosphere for 5 h. Cells were centrifuged at 8000 rpm for 10 min and re- suspended in 300 μL of BHI broth. An aliquot of 100 μL of cells was plated on BHI plates supplemented with 50 μg/mL of spectinomycin. The plates were incubated in a 10% CO_2_ incubator for 48 h [49]. Transformed cells containing plasmids integrated into the chromosome by a single homologous recombination event were selected on BHI plates supplemented with 50 μg/mL spectinomycin. Spectinomycin-resistant colonies were sub-cultured on BHI agar plates with no antibiotics. After sub-culturing for 3 days, the colonies were replicated on BHI agar with 1 mM IPTG. Spectinomycin-resistant clones were then grown in media without spectinomycin with the addition of 10% sucrose and IPTG to counter-select for double crossovers (the expression of *sacB* gene in pSJ13 makes the cells lethal in the presence of sucrose by forming fructo-polysaccharide). Colonies were streaked in BHI plates without antibiotics and BHI plates with 30 μg/mL of spectinomycin, and those that grew only on the former were selected [48,49]. The isolated recombinant plasmids with confirmed genomic deletion by PCR using the primers designed to amplify outside the 5′ and 3′ flanking regions were selected. This PCR product was also confirmed by sequencing. The resultant *apiA* isogenic mutant was designated as SJ13.

### 2.4. Immunofluorescence Microscopy

Growth and induction of *E. coli* harboring the plasmids were carried out as above. To immobilize cells for immunofluorescence assessment, 10 mL of each strain subculture was added into wells of the slide (Multitest Slide 8-Well, M.P Biomedicals LLC, Santa Ana, CA, USA) and air-dried. Ten microliters of primary antibody, anti-ApiA (0.7 mg/mL of rabbit polyclonal, Pocono Rabbit Farm and Lab, Canadensis, PA, USA), was added to each well at a 1:10 dilution in PBS and incubated for 2 h. After incubation, the slides were washed three times with PBS for 2–5 min each, and 10 μL of secondary antibody (1 mg/mL, goat anti-rabbit IgG FITC, Sigma-Aldrich, St. Louis, MI, USA) at a dilution of 1:80 in PBS was added and incubated for 1 h in the dark. After incubation, the slides were washed again three times with PBS for 2–5 min each and air-dried. The slides were then fixed using VECTASHIELD Antifade Mounting Medium (Vector Laboratories, Burlingame, CA, USA), covered with a coverslip, sealed, and examined and photographed using immunofluorescence microscopy (Confocal Imaging Facility, New Jersey Medical School, Newark, NJ, USA). Imaging was performed using Nikon A1R-A1 confocal microscope and Plan Apo VC 60× OIL NA-1.4 objective lens with 2.5× scanner zoom.

### 2.5. Electron Microscopy

Growth and induction of *E. coli* harboring the plasmids were carried out as above. Strains tested were pET-29b+ (negative control) and ApiA WT (full-length ApiA). Ten microliters of primary antibody, anti-ApiA (0.7 mg/mL of rabbit polyclonal antibody; Pocono Rabbit Farm), at a 1:10 dilution in PBS were added to bacterial cells and incubated for 2 h. Cells were washed three times with PBS and incubated with an immunogold-labeled secondary antibody (25 mg/mL, anti-rabbit IgG produced in goat and bound to gold particles (5 nm), Sigma-Aldrich) at 1:5 dilution for 1 h. Cells were washed with PBS and pelleted. The washed and pelleted cells were fixed with 2.5% glutaraldehyde (TED PELLA, Inc. Redding, CA, USA) for 2 h at RT and were washed again 3 times in PBS. Secondary fixation was carried out for 1 h with 1% osmium tetroxide (Sigma-Aldrich). The cells were again washed with PBS. A dehydration series was carried out with graded ethanol using 25%, 50%, 75%, and 100% ethanol each for 30 min. After the dehydration series, the samples were then transferred onto ultra-thin lacey carbon supported grid with 300 mesh (Sigma Aldrich) and imaged using a JEM-F200 transmission electron microscope (TEM) with a JEOL EDS detector and Gatan Oneview camera/DigiScan (Pleasanton, CA, USA). The images were taken under TEM (in transmission mode) at 200 kV and EDS under STEM mode (in scanning mode) at the Otto H. York Center for Environmental Engineering and Science (YCEES), New Jersey Institute of Technology (NJIT).

### 2.6. Auto-Aggregation Assay

Growth and induction of bacterial strains that included the empty plasmid and the full-length *apiA* gene with all deletions were carried out as previously described (see Figure 1). Quantitative assessment of the degree of auto-aggregation was determined by measuring the optical density of the supernatants at various time points. All strains were incubated in a shaker held at 37 °C for various time intervals after induction. Time 0 represents the OD 600 nm at time t = 45 min after induction. The OD 600 nm of the supernatant was measured using a NanoDrop One spectrophotometer (Thermo Fisher Scientific, Waltham, MA, USA) for 0, 35, 45, and 60 min following the induction period.

### 2.7. Buccal Epithelial Cell Culture

The TR146 human immortalized buccal epithelial cell line (10032305, Sigma-Aldrich) was grown in cell culture flasks (25 cm^2^ Corning, NY, USA) using complete growth media (HAM’s-F12 media supplemented with 10% of heat-inactivated fetal bovine serum (FBS; Gibco by Life Technologies, Grand Island, NY, USA) and 1X of Pen Strep Glutamine (Gibco by Life Technologies) at 37 °C with 5% CO_2_. When cells reached approximately 85–90% confluency, the growth media were discarded, cells were washed with PBS, and they were detached by the addition of pre-warmed 0.05% trypsin-EDTA-1X (Gibco by Life Technologies). Equivalent volumes of pre-warmed complete growth medium were added, and cells were transferred to a 15 mL conical tube and pelleted by centrifugation at 200× *g* for 10 min. The pelleted cells were resuspended in 1 mL of pre-warmed complete growth medium. Cells were enumerated using the automated cell counter (Countess; Thermo Fisher Scientific).

### 2.8. Epithelial Cell Binding by Thymidine-Radiolabeled Bacteria

ApiA constructs, including the positive full-length *apiA* control and the negative empty plasmid control (pET29b+) and all the *apiA* deletions (Figure 1), were grown and induced as described above, with the addition of 10 μL 2′DeoxyThymidine 5′triphosphate and tetra Na salt [Methyl-3H] (1 mCi/mL, PerkinElmer, Waltham, MA, USA) to the overnight culture of buccal epithelial cells (BECs), which were prepared as described above. Radiolabeled bacterial cells (250 μL of the 3 mL subculture and induced cells) and BECs (250 μL) were combined and incubated for 5 min at 37 °C on a rotor shaker. The mix was pelleted for 2 min at 150× *g*, and the pellet was washed twice using PBS and centrifuged for 2 min at 150× *g* to remove all unbound bacteria. The centrifugal speed allowed unbound bacteria to stay in suspension while pelleting the BECs. The pellet was resuspended in 200 μL of 0.1% Triton X and transferred to scintillation vials (Fisher Scientific), which were filled with Ecosiant A Scintillation solution (National Diagnostics, Charlotte, NC, USA). The amount of radioactivity was measured using an LS 6500 multi-purpose scintillation counter (Beckman Coulter, Brea, CA, USA) in the Office of Radiation Safety at Rutgers University. For all strains (Figure 1), the assay was carried out in technical quintuplicates. The entire experiment was repeated independently three times.

### 2.9. Complement Resistance Assays

Complement resistance assays were performed for both the *E. coli* strains expressing all *apiA* variants as well as an *A. actinomycetemcomitans* IDH781 wild-type and *apiA*-deleted strain of IDH781. For all the *E. coli* variants (Figure 1), cells were grown and induced as described above. The bacterial strains were incubated with normal human serum (NHS; Sigma Aldrich) and heat-inactivated human serum (HIS; Sigma Aldrich). For *E. coli* cells, a bacterial suspension of 10 μL was added to either 200 μL of 5% NHS or 5% HIS and incubated for 2 h at 37 °C. After incubation, 100 μL of each sample was serially diluted and plated on LB agar supplemented with 30 μg/mL kanamycin. Colony-forming units (CFU/mL) were calculated.

For *A. actinomycetemcomitans* experiments, wild-type IDH781 strain and the *apiA*-deleted IDH781 strain (SJ13) were initially grown on BHI agar supplemented with 0.6% yeast extract (Beckton Dickinson (BD), Franklin Lakes, NJ, USA), 0.8% dextrose, and 0.4% sodium bicarbonate. Colonies were picked from plates and checked for purity. Plates were scraped to obtain sufficient starting cultures and then inoculated into BHI broth (Beckton Dickinson) supplemented with 6% yeast extract (BD). Cells were subjected to centrifugation for 10 min at 4000 rpm, washed with PBS, and re-suspended to achieve an OD_600nm_ of 1.00. An aliquot of 10 µL from each strain (IDH781 and SJ13) was subjected to tests in the presence of a control where no human serum was used and grown for 2 h under anaerobic conditions. In a second set of experiments, both IDH781 and SJ13 were tested either in the presence of 50% normal human serum (NHS) or in the presence of heat-inactivated serum (HIS) and grown for two hours in an anaerobic chamber [29]. All samples were serially diluted and plated onto BHI agar plates for colony enumeration. IDH781, IDH781HIS, SJ13, and SJ13HIS results were normalized to cells in HIS as compared to NHS.

### 2.10. ELISA to Identify the ApiA Passenger Domain That Binds to Recombinant Factor H

Growth and induction of all the various *apiA* deletions and controls were performed as previously described in this study. One ml of bacterial cell suspension was incubated with 2 µg/mL of human FH (Complement Technology Inc., Tyler, TX, USA) for 30 min at 37 °C with vigorous shaking (850 rpm). The bacterial suspension was pelleted and washed three times using PBS then re-suspended in PBS, and the final concentration was adjusted to 10^8^ bacteria/mL. From this bacterial suspension, 60 μL were transferred to 96-well enzyme-linked immunosorbent assay (ELISA) plate wells (Nunc PolySorp, Thermo Fisher Scientific), which were allowed to dry overnight at 37 °C. The ELISA plate wells were washed three times with PBS, blocked with 100 μL of 5% skim milk in PBS for 1 h at 37 °C. The wells were washed again three times with PBS and incubated with 50 µL of anti-FH monoclonal antibody (1 mg/mL, EPR6225, Abcam, Cambridge, MA, USA) diluted at a ratio of 1:500 in 5% skim milk for 1 h at RT. Wells were washed three times with PBS and incubated with HRP-conjugated goat anti-rabbit super clonal antibodies (0.0625 µg/mL, Thermo Fisher Scientific) for 1 h at RT. The wells were washed again 3 times with PBS, and 50 µL of TMB (3,3′,5,5′-tetramethylbenzidine) substrate (Thermo Scientific Pierce 1-Step Ultra TMB ELISA Substrate) solution was added. After 10 min of incubation, the reaction was stopped using 2 M H_2_SO_4_, and the absorbance at 450 nm was determined using a Tecan plate reader. Experiments were repeated three times in duplicate.

### 2.11. Peptide Effects on Complement Sensitivity Due to Factor H Binding

Peptides were designed to target the D101-217 amino acids of ApiA (Figure 1; EZBiolab, Carmel, IN, USA). This peptide was 85 amino acids in length, and it was divided into 3 peptides 25–30 amino acids in length. A second peptide set (Biomatik, Wilmington, DE, USA) consisting of 32 amino acids was designed to replicate the deleted sequence in variant D186-217, because it did not bind to FH in the ELISA assay, suggesting that this region was critical for FH binding. To test the ability of these peptides to affect complement activity, *E. coli* BL21 with pET21b+ was grown and induced as previously described. The peptides 1–6 (1 mg/mL) were incubated with 5% NHS for 1 h at 37 °C with rotation. Bacterial cells were then washed, added to this mixture, and incubated for 2 h at 37 °C to determine if the peptides added to serum were binding to C3b and FH, which would reduce their availability to attack the plasmid-containing bacteria. This would reduce the sensitivity of the strain to complement. The cells were serially diluted and plated as previously described. As a control, bacterial cells with no peptides or serum were also included. Experiments were performed in triplicate and repeated three times.

### 2.12. Growth Conditions for RNA Extractions

Cells were grown using two growth conditions: (1) IDH781 and IDH781Δ*apiA* (SJ13) in the absence of serum were grown on BHI agar plates and grown under anaerobic conditions (10% CO_2_, 10% H_2_, and 80% N_2_) for 48 h, and (2) IDH781 and IDH781Δ*apiA* were exposed to 50% NHS or 50% HIS for 2 h under anaerobic conditions. Cells were collected from plates, re-suspended, grown in BHI broth, and then used for RNA extractions, as outlined below. Cells were uniformly distributed by vigorous vortexing, and OD_600_ was measured. The strains were subjected to 50% NHS or were grown in the absence of serum for 2 h (under anaerobic conditions). Cells were collected for RNA extraction as outlined below.

### 2.13. RNA Extractions and qRT-PCR

RNA was harvested from two growth conditions described above: (1) IDH781 and IDH781Δ*apiA* grown on BHI agar plates with no serum added for 48 h; and (2) IDH781 and IDH781Δ*apiA* exposed to 50% NHS or no serum for 2 h. Cells were collected from wild-type IDH781 and SJ13 in BHI broth. RNA isolation was carried out as previously described [13,50]. To stabilize the bacterial RNA, ice-cold 0.9% saline supplemented with 1/10th volume of 95% ethanol and 5% citric acid saturated phenol (Sigma Aldrich, Burlington, MA, USA) was added. The cells were centrifuged at 10,000 rpm for 5 min at 4 °C, and the cell pellets were flash-frozen in liquid nitrogen and stored at −80 °C until further use. RNA was isolated from the *A. actinomycetemcomitans* strains by the hot phenol method with the following modifications [50]. Glass beads were added to frozen pelleted cells followed by the addition of 700 μL ice-cold suspension buffer (30 mM sodium acetate at pH of 4.3, containing 1% β-mercaptoethanol, 2 mM aluminum ammonium sulfate, and 2 mM EDTA (Sigma Aldrich)). The cell suspension was bead-beaten (Biospec Products, Bartlesville, OK, USA) for 10 s to make sure the cells were dispersed evenly. A volume of 102 μL of preheated (65 °C) lysis buffer (300 mM sodium acetate at pH of 4.3, 10% β-mercaptoethanol, 8% SDS, and 16 mM EDTA at pH of 8.0) was added, bead-beaten for 20 s, and incubated at 65 °C for 3 min. An equal volume of preheated acidic phenol saturated with citrate buffer at pH of 4.0 (65 °C) was added and bead-beaten for 20 s (5Xs) with 1 min intervals maintaining the temperature at 65 °C. The phenol mixture was centrifuged at 14,000 rpm for 20 min at 4 °C. After repeating acidic phenol extraction, the aqueous phase was extracted with ice-cold chloroform (Sigma Aldrich) (2 times). The RNA was precipitated with the addition of 10% sodium acetate and 100% ethanol (2.5 volumes). The ethanol precipitated RNA was pelleted down by centrifugation at 14,000 rpm for 20 min and washed twice with 70% ethanol. The RNA pellet was air-dried and re-suspended in RNAse-free water. Total RNA was quantitated using a NanoDrop One Lite spectrophotometer (Thermo Fisher Scientific). The quality of the RNA was determined by the ratio of absorbance at 260/280 nm. To improve the quality of the total RNA, samples were passed through Micro Bio-Spin P-30 Gel Columns (Bio-RAD, Cat No. 732-6250).

After the gel filtration step, RNA samples were quantitated, and 500 ng of total RNA was mixed with RNA sample loading buffer (Sigma Aldrich), denatured at 65 °C for 10 min, ice-cooled, and analyzed on 1% TAE agarose gel. The RNA integrity was confirmed by visualizing the staining intensity difference between 23S and 16S rRNA and using the Agilent TapeStation system (Molecular Resource Facility, Rutgers, Newark). The purified total RNA samples were stored at −80 °C until further use. The total RNA (5 μg) was subjected to DNase I treatment to remove the contaminant genomic DNA using an RNA purification kit (Zymo Research, Irvine, CA, USA). As a negative control, complete DNA removal was confirmed in all the samples by PCR using *apiA* primers (Table 2) before subjecting the samples to qPCR.

### 2.14. Quantitative RT-PCR

cDNA was obtained from the isolated DNA-free RNA using a high-capacity reverse transcription kit (Applied biosystems, CA, USA) according to the manufacturer’s instructions. One µg of RNA was converted into cDNA. qPCR reactions were performed in the CFX Opus 96 Real-Time PCR System (BIO-RAD) using PowerUp SYBR green master mix (Applied Biosystems, Carlsbad, CA, USA). Twenty-five microliter reactions were performed each time. cDNA was used in a dilution of 1:250 for reference gene 5SrRNA. Initial denaturation was performed at 94 °C for 10 min followed by 40 cycles of amplification (94 °C, 20 s; 56 °C, 20 s; and 72 °C, 20 s). Specificity of the products was assessed by melting curve analysis [13,49,50]. Negative control reaction with no reverse transcriptase was included in each run. Data were analyzed using Bio-Rad CFX Maestro software version 2.0 (Cambridge, MA, USA). The differential gene expression between IDH781 and SJ13 strains was calculated based on 2^−ΔΔCт^ value compared to 5S rRNA using three biological replicates, each of which had three technical replicates. The results were subjected to Student’s *t*-test for statistical significance (*p* < 0.05).

### 2.15. Statistical Analysis

All experiments were performed on at least three separate occasions, with two or three technical replicates in each experiment. Statistical analysis comparing data was performed using one-way ANOVA for statistical significance with a confidence interval of 5% (*p* < 0.05) and Tukey–Kramer HSD for pairwise comparisons (JMP software package, version 12.0.1). Student’s *t*-test was performed to compare the qRT-PCR results, and Bonferroni corrections were applied.

## 3. Results

### 3.1. Immunofluorescence and Transmission Electron Microscopy

**Figure 2:**
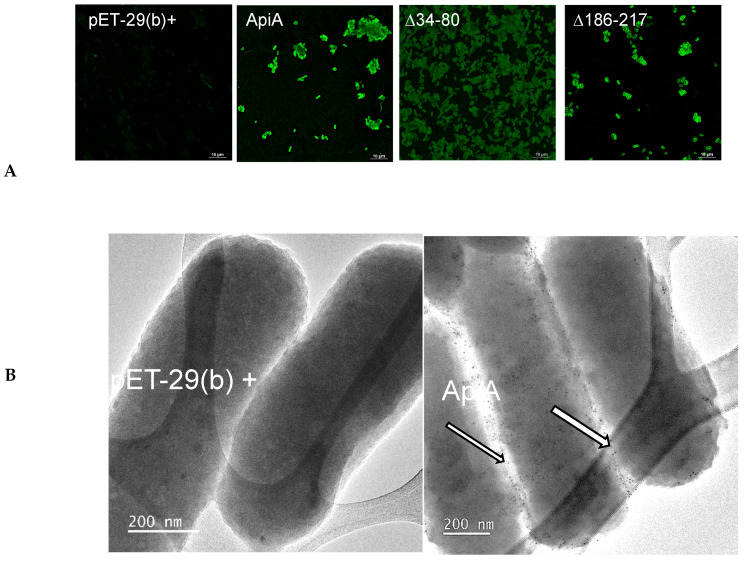
Immunofluorescence and TEM showing membrane labeling. Upper panels (left to right): pET29b+ empty plasmid, full-length ApiA (apple green), D34-80, and D186-217 (apple green) (**A**). Transmission electron microscopic images of immunogold particles are shown in right panel (**B**) (see arrows).

To confirm the surface expression of ApiA and variants of *E. coli*, antibody staining was performed using a polyclonal anti-full-length ApiA antibody. The control strains, BL21 with pET21b+, failed to be recognized by the antibody. In contrast, full-length *apiA,* ∆34–80, and D186-217 showed immunofluorescence. The remaining gene regions expressed in *E. coli* failed to show immunofluorescence. To further assess the surface expression of ApiA in *E. coli*, TEM was used to examine the BL21 strains with pET21b+ (the negative control) and the ApiA full-length gene. The TEM results showed electron-dense immunogold areas in the full-length ApiA on the outer membrane as compared to the E. coli strain containing the empty plasmid, which failed to show immunogold labeling (Figure 2B).

### 3.2. Auto-Aggregation Assay

A quantitative assessment of the auto-aggregation of the *E. coli* strains was performed based on the optical density measurements over 45 min post-induction (Table 3). The higher the optical density in the supernatants indicates the inability of the bacteria to auto-aggregate since the supernatants remain in a homogenous solution. The control strain, BL21 with pET21b+, failed to aggregate. Full-length ApiA and ApiA WT showed a significant change in the optical density and precipitated to form a pellet at the bottom of the test tube. ∆81–100 and ∆186–217 displayed similar results to those of full-length ApiA, indicating the missing region had no effect on their ability to auto-aggregate. ApiA variants ∆28–33, ∆34–80, ∆101–185, ∆81–185, and ∆28–185 showed reduced auto-aggregation. These results suggest the aggregation domain is contained in regions 28–80 and 101–185 of the passenger domain, which is feasible given the repeat regions located at 26–73, 74–122, and 148–184 (Figure 1).

### 3.3. Buccal Epithelial Cell Binding by Thymidine-Radiolabeled Bacteria

**Figure 3:**
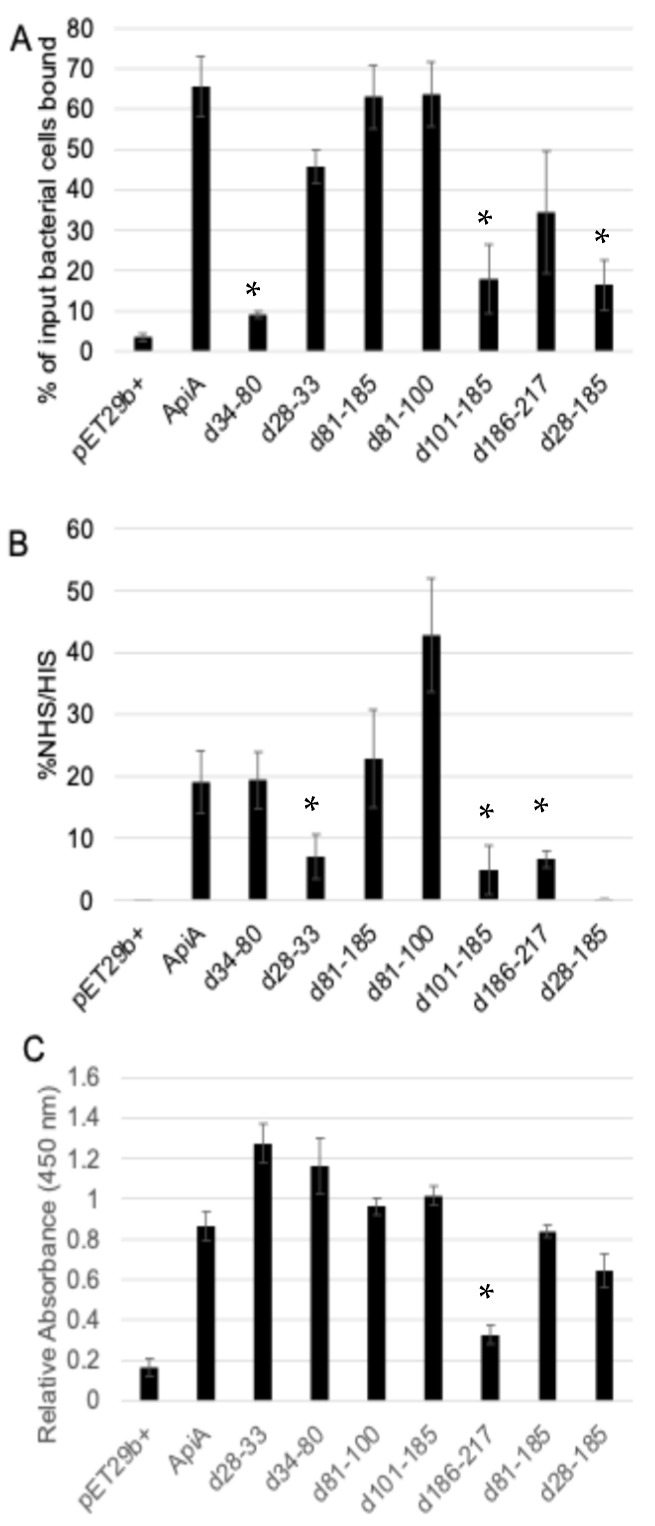
Buccal epithelial cell binding (**A**). Serum survival = % survival of the various strains when treated with 5% NHS and 5% HIS. (**B**). Factor H binding (**C**). ELISA was used to determine if *E. coli* strains treated with Factor H had the ability to interact or bind with Factor H. * represents *p* ≤ 0.01.

Epithelial cell binding was assessed by analyzing radioactively labeled bacteria and their ability bind to BECs. The percentage of binding was calculated by measuring the ratio of the radioactive counts per minute of bound bacterial cells to the total bacterial input counts per minute. As expected, the negative control, BL21 with pET21b+, failed to bind (Figure 3A). Variant ∆34–80 displayed the lowest level of binding, indicating the BEC binding site is likely within that region. pET29b+ (the empty plasmid vector) showed the lowest level of binding, while the full-length ApiA showed high levels of binding. The lowest binders were the empty plasmid, ∆34–80, ∆101–185, and ∆28–185. The most relevant amino acid deletions that contribute to binding therefore were ∆34–80 and ∆101–185.

### 3.4. Complement Resistance Assays

A quantitative assessment by CFU/mL plating for the complement resistance of the *E. coli* strains was performed by evaluating their survival in 5% normal human serum (NHS) relative to heat-inactivated serum (HIS). The negative control, BL21 with pET21b+, and ∆28–185 did not survive in the presence of NHS; however, *apiA*, D34-80, D81-185, and D81-100 were not sensitive to the serum. The results are shown in Figure 3B.

**Figure 4:**
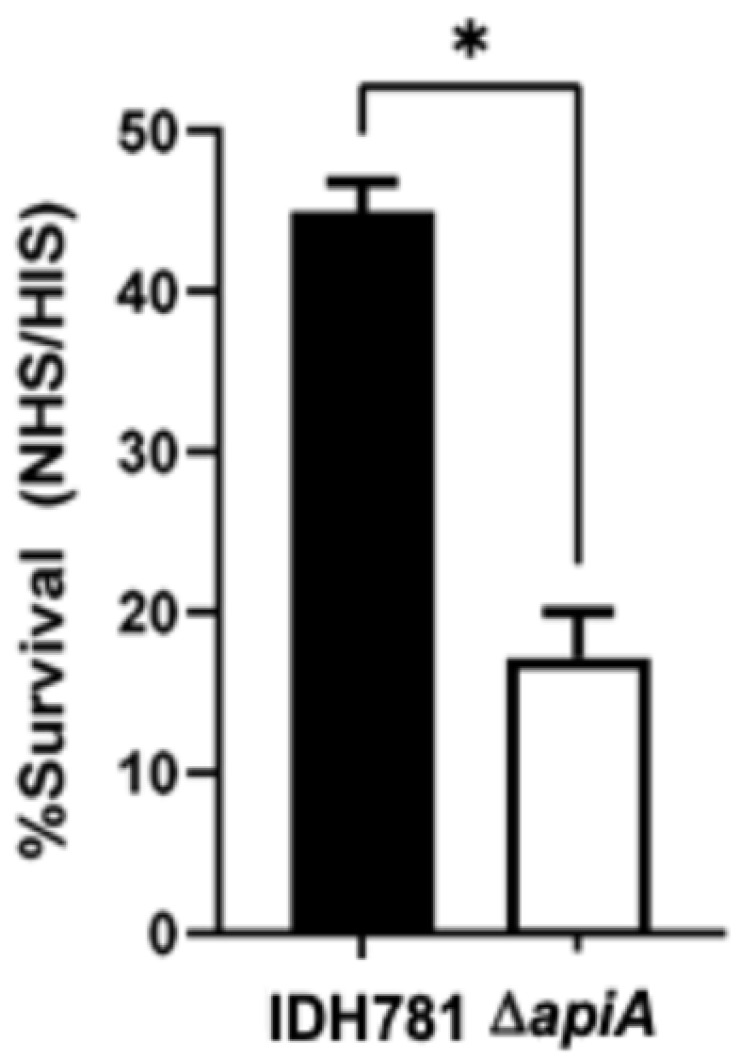
*A. actinomycetemcomitans*’s sutvival in serum showing ratio of surivival in normal serum as compared to heat-inactivated serum. * indicating significant difference (*p* ≤ 0.05).

As per Figure 3B, IDH781 and the *apiA* deletion strain (SJ13) were also evaluated for their complement resistance, and IDH781 showed 45% resistance while SJ13 showed 17.2% resistance in comparison to the IDH781 control as determined by the CFU/mL plating of cells exposed to 50% NHS and HIS under anaerobic conditions (*p* ≤ 0.05) (Figure 4). IDH781 is a well-maintained serotype d rough strain, and its growth was under anaerobic conditions, which might result in differences between our study and other studies [15,29,35].

### 3.5. ELISA to Identify the ApiA Passenger Domain That Binds to Recombinant Factor H

The proposed mechanism of serum resistance is through the binding of the regulatory protein Factor H. Here, using an ELISA, recombinant variants were immobilized and assessed for their ability to bind purified Factor H (Figure 3C). Full-length ApiA and the variants ∆28–33, ∆34–80, ∆81–100, ∆101–185, ∆81–185, and ∆28–185 showed binding to Factor H. Variant ∆186–217 displayed a reduced binding ability in comparison to the other strains and the positive full-length ApiA, indicating the binding region is likely within amino acids 186–217. The control strain, BL21 with pET21b+, failed to bind.

### 3.6. Peptides’ Effects on Complement Sensitivity

**Figure 5:**
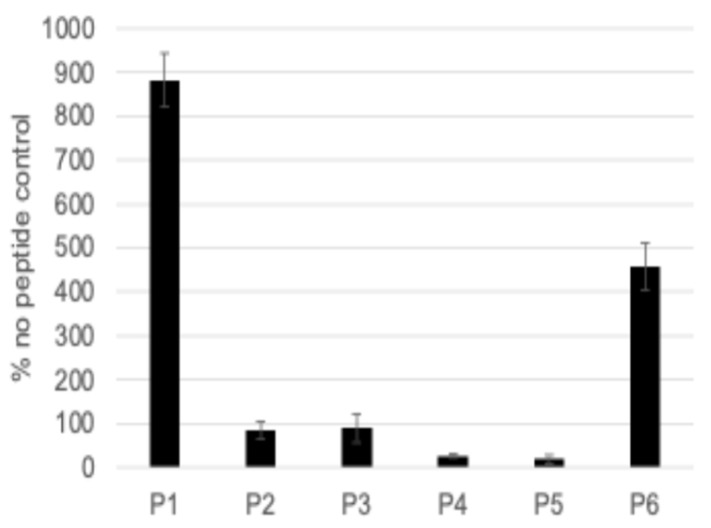
Indirect measurement of peptide binding to Factor H. Higher bacterial survival in the presence of peptides (1–6), the higher the level of interference with FH availability.

The addition of peptide P1 to the pETb+ empty plasmid control strain showed a nearly 900-fold increase in survival over that of the serum with the no-peptide-added control (Figure 5). In contrast, the addition of peptides P2, P3, P4, and P5 showed no significant difference in colony-forming units as compared to the control. The addition of peptide 6 provided the cells with about a 450-fold increase in survival in the presence of serum as compared to when no peptides were added (Figure 5).

### 3.7. Quantitative RT-PCR of Cells Grown with and Without Serum

**Figure 6:**
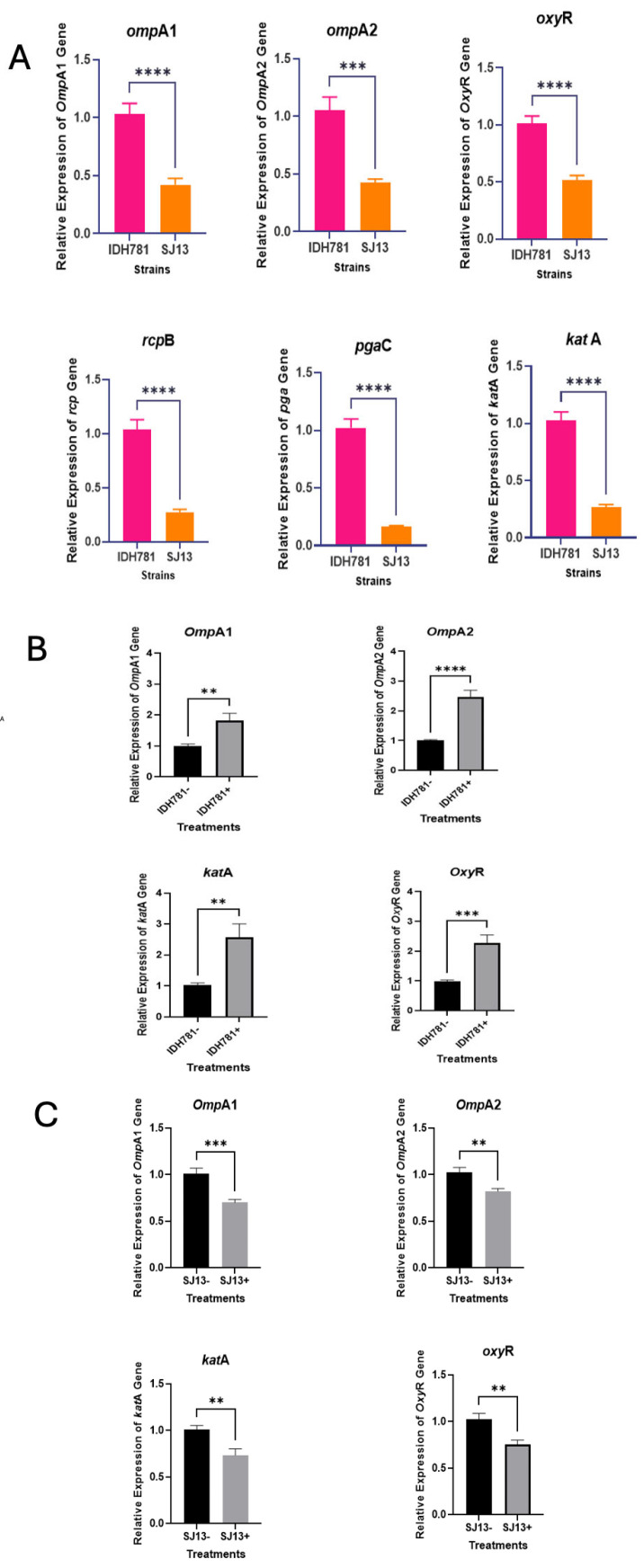
qRT-PCR assessment comparing expression of specific genes in the absence or presence of serum. These assessment were made in the absence of any serum treatment (**A**). The next group compared serum treatment to no treatment in, IDH 781 (**B**). And the final comparison was in SJ13 (IDH with an *apiA* deletion comparing serum treatment to no serum treatment (**C**). Note that stars indicate significant differences at a minimum of (*p* ≤ 0.05).

The relative expression of selected genes like *omp*A1 and *omp*A2, which are responsible for conferring complement resistance, were analyzed to gain a better understanding of how the deletion of *apiA* would affect the expression of these genes. qRT-PCR was used to assess the expression levels of genes involved in attachment and biofilm formation, like *rcp*B and *pga*C, as well as genes like *oxy*R and *kat*A, which are critical for stress resistance. In the first experiment WT-IDH 781 was compared to SJ13 (IDH with the *apiA* deletion. Here IDH 781 showed elevation for all genes tested in the absence of serum (Figure 6A). In the second set of experiment both WT-IDH 781 strain and SJ13 (IDH with the *apiA* deletion were tested in the presence or absence of serum (Figure 6B,C). Here IDH781 consistently showed elevation in expression of the genes assessed (Figure 6B) while SJ13 consistently showed a depression in gene expression in the presence of serum (Figure 6C). The significance levels were calculated by means of a Student’s t-test and showed that the level of difference reached (*p* < 0.001) when the wild-type strain was compared to the apiA-deleted strain SJ13.

The relative expression levels of the selected genes, *ompA1*, *ompA2*, *katA,* and *oxyR,* were assessed in response to serum treatment in wild-type IDH781 (IDH781+ = serum added; IDH781 = no serum added) using qRT-PCR for the expression levels of genes. A similar assessment was conducted with the Δ*apiA* strain (SJ13), and the qRT-PCR showed that all the assessed genes were upregulated in IDH781 in the presence of serum and downregulated in the SJ13 *apiA*-deleted strain (Figure 6B). All reading showed significant differences in their expression profiles of *oxyR* (*p =* 0.01), *kat*A (*p =* 0.01), *omp*A1 (*p =* 0.01), and *omp*A2 (*p =* 0.01)

## 4. Discussion

*A. actinomycetemcomitans* contributes to periodontitis in both the early and later stages of the disease process [51]. The colonization of teeth and soft tissue occurs early in life, and *A. actinomycetemcomitans* utilizes many strategies to colonize soft tissues prior to tooth eruption [52,53]. Key among the adhesins known to influence these binding characteristics is the widespread colonization island of genes responsible for fimbrial structures and extracellular polysaccharides that lead to non-specific binding to abiotic surfaces such as enamel [8]. In addition, Aae and ApiA are two autotransporter proteins that enable *A. actinomycetemcomitans* to bind to soft tissue such as the gingival epithelium [14,15,54]. These two omps have very different structures, with Aae being monomeric and non-aggregating, and ApiA being trimeric with auto-aggregation or clumping [16]. Efforts to gain a better understanding of ApiA’s diverse functional activity have been difficult largely because auto-aggregation complicates accurate quantitative assessments. Clumping has been shown to be associated with the C-terminus of ApiA, and as a result, a hybrid protein was created that merged the C-terminus of the monomeric form of Aae to the passenger domain of trimeric ApiA. Using this strategy, cellular clumping was minimal, which allowed for the exploration of soft tissue binding, auto-aggregation, and biofilm formation in the fused protein expressed in an *E. coli* host [16]. Sequential deletions were created in the passenger domain, and each deletion was examined for its effect on auto-aggregation, tissue binding, and biofilm formation. These experiments led to the conclusion that the C-terminus of ApiA was required for trimerization, auto-aggregation, and biofilm formation, although it was possible that the gene-deleted regions as expressed in the *E. coli* host in the monomeric protein were not truly representative of the functional activities exhibited in the trimeric autotransporter protein. Nevertheless, the results of the hybrid fusion experiments were helpful in efforts to re-examine deleted areas in the N-terminal passenger domain of the trimeric autotransporter, especially in the case of highly relevant complement resistance and Factor H binding [16]. The impact that *A. actinomycetemcomitans* has on the damage/response process can be likened to AIDS, in that its effect on the host dampens the immunological response, permitting other microbes to persist and thrive in an immunologically compromised host domain [55,56]. In the early stages of disease, *A. actinomycetemcomitans* has an impact on the innate immune system by virtue of its effect on (1) the epithelial barrier (cytolethal distending toxin [57]), (2) serum-derived complement resistance (Omp and ApiA) [38], and (3) leukocytes and monocytes (leukotoxins) [58,59]. The least understood of these virulence traits is complement resistance, a trait that occurs in the earliest stages of disease [17]. This study has been developed to better understand the role of ApiA as a trimeric autotransporter protein in complement resistance as well as auto-aggregation and epithelial cell binding [15,16].

ApiA, a trimeric multifunctional protein, has been proposed to be a critical virulence factor in periodontitis, occurring in children and young adolescents [15]. Most importantly, *A. actinomycetemcomitans* ApiA is thought to affect the early stages of disease in patients who suffer from Stage III Grade C (LAgP) periodontitis [16]. As a multifunctional omp, ApiA is uniquely positioned for auto-aggregation and biofilm formation as well as epithelial cell binding [18]. When *A. actinomycetemcomitans* migrates subgingivally, it is confronted by crevice fluid, a serum exudate containing complement protein [45]. ApiA was first characterized by Asakawa in 2003 [29]. While these studies have provided some insight into the importance of ApiA (e.g., Omp100), the specific identification of regions of interest in the gene locus and the functionality of the gene remained unresolved [15,17,29]. Defining these regions could help develop potent vaccine candidates and/or peptides related to active sites that could be used to interfere with local complement activity.

This study has helped identify regions within the passenger domain of *apiA* that are critical for the multi-functional capability of ApiA. Disease progression is not caused by microorganisms alone but is due to the way in which the host immune response modulates the challenge, which could either amplify or reduce disease progression [27,33,60,61]. Microbes are known to protect themselves from complement-related cell surface destruction by hijacking host complement regulators from plasma or other body fluids [25]. Pathogens use a range of strategies that allow them to survive and disseminate in the host. A strategy of immune evasion through molecular mimicry can provide bacteria with the ability to imitate host surface proteins, permitting them to persist within the host so as to avoid destruction by complement proteins [62,63]. *A. actinomycetemcomitans* displays serum resistance and immune evasion by binding to the complement protein FH by means of its outer membrane protein, ApiA [46]. Essentially, sequestering Factor H allows the bacterium to masquerade as a mammalian cell so as to avoid clearance via complement activity [27]. It is generally hypothesized that upon binding Factor H, the alternative complement pathway is downregulated, promoting host innate immune evasion [61]. Tricking the complement system, particularly the alternative complement pathway, enhances microbial virulence and is spontaneously activated on non-protected surfaces that would be vulnerable. Many bacterial pathobionts like *Neisseria meningitidis, Yersinia pestis,* and *Treponema denticola* have a variety of strategies to mimic host cell surface molecules, like heparin or glycosaminoglycan, which cause the complement regulator protein FH to bind to their outer membrane protein, thereby downregulating the complement system in order to allow for survival in the host [64,65,66].

Over years of evolution, FH binding as developed in human cells has been replicated in bacterial cells such that there is now competition for the sequestration of FH between specific bacterial cells and host cells [65]. Identifying the domain required for the interaction with FH could help develop substances (such as small peptides) to help to strengthen the resistance to complement sensitivity. This study identified ApiA domains responsible for binding to buccal epithelial cells, auto-aggregation, and the domain that is responsible for serum resistance. The examination of *A. actinomycetemcomitans*-related ApiA expression and functionality in an *E. coli* host has enabled the identification of regions responsible for surface expressed proteins that can be induced, isolated, analyzed, and functionally characterized. The focus of this study was on the surface expression of ApiA. Auto-aggregation and buccal epithelial cell binding can only take place if surface interactions occur. To ensure that these functional activities were due to the surface expression of ApiA, fluorescent antibody detection, followed by a TEM examination of gold-labeled antibody directed to the passenger domain of ApiA, was carried out. These tools provided visual evidence that ApiA was surface-expressed. After assurance that the surface expression was reproducible, the functional activities of surface expressed proteins were examined using the quantitation of auto-aggregation and epithelial cell binding. In all cases, ApiA expression proteins induced in *E. coli* showed distinctive and quantitatively reproducible functionality. ApiA binding to FH was used to select the most likely region of the *apiA* gene responsible for complement resistance and FH binding. It was revealing to discover that polyclonal antibody and a particular monoclonal antibody proposed to detect FH had little to no specificity and, as such, bound to all cell surfaces (including a host containing an empty plasmid that failed to express surface proteins). In contrast, one monoclonal antibody bound to the full-length ApiA as expressed in *E. coli* as well as to a very specific region containing 32 amino acids. To confirm the specificity of binding to the complement region of interest, peptides associated with the critical 32 amino acid region were made and then three peptide sequences within this 32-amino-acid region were designed. These peptides were added to untreated serum containing complement protein and revealed that a particular sequence containing a set of 13 amino acids blocked serum-related complement activity. This completed our efforts to identify the *apiA* region of interest in *E. coli.* Our attention was then focused on deleting the *apiA* gene in *A. actinomycetemcomitans* IDH 781 to determine its effect on complement resistance as well as how this deletion affected the expression of several auxiliary genes that regulated the expression of genes of functional prominence.

Reacting IDH781, the *A. actinomycetemcomitans* WT strain, with serum resulted in 45% serum resistance when compared to HIS and 38% when compared to the *apiA*-deleted strain (SJ13). Other recent studies suggested that OmpA-1 could provide added protection against complement proteins [17]. Studies have also shown that membrane vesicles secreted by strains of *A. actinomycetemcomitans* can also contribute to complement resistance [38,51]. These forays into complement resistance in *A. actinomycetemcomitans* indicate that this form of immune avoidance is potentially greater than currently imagined. In addition, experiments have not been extended to serotype b JP2 strains, most likely because of cloning difficulties in serotype b strains [67,68]. In spite of these shortcomings, the results of *A. actinomycetemcomitans* serotype a and d strains, strains which are not related to disease and which were grown in a manner in the laboratory that may have limited their translatability to their real-world activity, our data provide a good starting point. Also, the fact that the isolation of membrane vesicles from *A. actinomycetemcomitans* shows complement activity supports the concept that this activity can be more widespread in vivo [17,38]. Further, our focus on these restricted experimental conditions was due to the fact that previous studies have failed to make headway in ApiA-related complement resistance. Within the confines of the limitations of this study design, we feel that the data as presented provide a good starting point for future investigations.

The second aim of this study was to determine whether there were genes that were co-regulated with *A. actinomycetemcomitans apiA* in its interaction with serum. Using qRT-PCR, a series of candidate genes were examined, which included *ompA1*, *ompA2*, *oxyR,* and several other genes responsible for homeostatic equilibrium. For example, when *ompA1* and *ompA2* gene expression was assessed in wild-type IDH781 as compared to the *apiA*-deleted strain (SJ13) in the absence of serum, there was an approximate increase in gene expression by 50% in the wild-type strain. In comparison, *katA* and *OxyR* and *ompA1* and *ompA2* were all upregulated in the presence of serum, while these genes were all downregulated in the presence of serum in SJ13, suggesting that the *apiA* gene is not just responsible for serum sensitivity but could also be responsible for other more globally regulatory gene responses.

Overall, these data suggest that not only does the increased expression of *apiA* in the presence of serum result in a reduction in serum sensitivity for itself and its community partners, but this response to serum also provides *A. actinomycetemcomitans* with added ways of avoiding environmental hazards by upregulating genes for biofilm formation, attachment, and oxygen resistance. While much more work is required, these interactions imply that a multifaceted/coordinated response is a pre-requisite for life in a complex ecological environment. *A. actinomycetemcomitans* appears to possess many ways of addressing its need for adaptability, including leukotoxin, cytolethal distending toxin production, and complement resistance, but extrapolation from the data presented above suggests that many other interactions are required for survival in a complex ecosystem and that these unanswered questions warrant continued and expanded research.

Several limitations of this study are clear and include but are not limited to the following: (1) the need to assess varying growth media, (2) the need to assess different stages of growth, and (3) the need to assess various strains and species of *A. actinomycetemcomitans*. In the future, the JP2 serotype b strain of *A. actinomycetemcomitans* could be examined. Since the goal in this study was to provide initial data, future studies could use site-directed mutagenesis once the critical amino acids required for various functions related to *A. actinomycetemcomitans* survival have been identified. In addition, a more precise definition of FH binding could determined by using alanine substitutions in the regions of the 13-mer amino acid that has been found to be responsible for Factor H binding in this study. Furthermore, data related to peptide 1 provide clues that ApiA may be involved in additional ways, interfering with complement pathways such as the lectin or classical pathways [24,69]. For example, elevated levels of ompA1 and ompA2 in the presence of serum support the work by Lindblom and associates and can potentially represent added ways in which *A. actinomycetemcomitans* shows adaptive capabilities [17,69].

## 5. Conclusions

(1) Studies designed to examine the specific region in the *A. actinomycetemcomitans apiA* gene responsible for complement resistance were assessed using an *E. coli* vector to examine its complement resistance. Sequential gene deletions in *apiA* were examined by immunofluorescence and immunogold transmission electron microscopy for surface expression and were confirmed by measuring auto-aggregation and buccal epithelial binding to assess the functional surface expression of *apiA*;
(2) *E. coli*-deleted regions (∆34–80 and ∆186–217) failed to show epithelial cell binding (∆34–80) and complement resistance (∆186–217);
(3) Factor H binding, critical for complement resistance via the alternative pathway, was used to probe the region(s) most likely responsible for complement resistance, and a 32-amino-acid protein within the ∆186–217 deletion was identified;
(4) Peptides were designed for further testing within this 32-amino-acid region, and a 13-amino-acid segment provided preliminary evidence that this area was responsible for complement resistance;
(5) *apiA* was deleted in *A. actinomycetemcomitans* IDH781, and qRT-PCR was used to identify several other relevant genes in *A. actinomycetemcomitans* that were either up- or downregulated in the presence or absence of serum in wild-type *A. actinomycetemcomitans* or in Δ*apiA. actinomycetemcomitans.* It was proposed that *apiA* could be associated with global regulation or some other regulatory manner that could affect the expression of prominent stress-related genes that could play a role in overall *A. actinomycetemcomitans* adaptability and stress survival;
(6) This is the first study to identify a specific region within *apiA* responsible for complement resistance via the alternative pathway and, as such, provides a good starting point for future studies that can achieve a more in-depth model of complement resistance and the role of *apiA* in the global regulation of *A. actinomycetemcomitans*.

## Figures and Tables

**Table 1:** Bacterial strains and plasmids.

*E. coli* Strains	Relevant Characteristics	Reference or Source
NEB5α	*fhuA2 Δ(argF-lacZ)U169 phoA glnV44 Φ80Δ (lacZ)M15 gyrA96 recA1 relA1 endA1 thi-1 hsdR17*	New England Biolabs
Mach1-T1^R^	F- φ80(*lac*Z)∆M15 ∆*lac*X74 *hsd*R(r_K_-m_K_+) ∆*rec*A1398 *end*A1 *ton*A	Invitrogen
Stellar	F–, *end*A1, *sup*E44, *thi*-1, *rec*A1, *rel*A1, *gyr*A96, *pho*A, Φ80d *lac*ZΔ M15, Δ(*lac*ZYA-*arg*F) U169, Δ(*mrr*-*hsd*RMS-*mcr*BC), Δ*mcr*A, λ–	Clontech
BL21(DE3)	*fhuA2 [lon] ompT gal (λ DE3) [dcm] ∆hsdS* *λ DE3 = λ sBamHIo ∆EcoRI-B int::(lacI::PlacUV5::T7 gene1) i21 ∆nin5 *	New England Biolabs
SJ100	BL21(DE3) containing plasmid pET29b (+)	This study
*A. actinomycetemcomitans* strains	Relevant characteristics	Reference or source
IDH781	Wild-type human *A. actinomycetemcomitans*, serotype d, spectinomycin-resistant	[47]
IDH781∆*api*A	Gene deletion of *apiA* in strain IDH781; SJ13	This study
Plasmid	Relevant characteristics	Reference or source
pJT1	Suicide vector, Spectinomycin-resistant	[48]
pET29b (+)	Expression vector, T7 promoter, Kanamycin-resistant	Novagen
pSJ101	pET29b+ containing full-length *apiA*; designated ApiA in text	[35]
pSJ102	pET29b+ containing truncated *apiA*; amino acids 28–33 deleted; designated ∆28–33 in text	This study
pSJ103	pET29b+ containing truncated *apiA*; amino acids 34–80 deleted; designated ∆34–80 in text	This study
pSJ104	pET29b+ containing truncated *apiA*; amino acids 81–100 deleted; designated ∆81–100 in text	This study
pSJ105	pET29b+ containing truncated *apiA*; amino acids 101–185 deleted; designated ∆101–185 in text	This study
pSJ106	pET29b+ containing truncated *apiA*; amino acids 186–217 deleted; designated ∆186–217 in text	[35]
pSJ107	pET29b+ containing truncated *apiA*; amino acids 81–185 deleted; designated ∆81–185 in text	This study
pSJ108	pET29b+ containing truncated *apiA*; amino acids 28–185 deleted; designated ∆28–185 in text	This study

**Table 2:** Primers used in this study.

Oligonucleotides	Sequence (5′→3′)	Source
Primers to amplify full-length *apiA*		
ApiA-NdeI-F	GGAATTCCATATGACATATCAATTATTTAA	[35]
ApiA-EcoRI-R	CGGAATTCTTACCACTCAAAGTTTAAACCG	[35]
Amino acid 28–33 deletion in ApiA to construct pSJ102		
DNF 2	GTCGATGCATTGGCTAAAGACTCTGCTAATCTTCCACAACAA	This study
DNR 2	TTGTTGTGGAAGATTAGCAGAGTCTTTAGCCAATGCATCGAC	This study
Amino acid 34–80 deletion in ApiA to construct pSJ103		
103F 2	GCTGAAAATCCTGGGGGGATCGATAGATTAGCTAAG	This study
103R 2	CTTTGCATTTCTATCGATCCCCCCAGGATTTTCAGC	This study
Amino acid 81–185 deletion in ApiA to construct pSJ107		
DNF 3	GTATAGAAAAAGATGTTATGCGTAACACTTTTAGATCTTCAAGC	This study
DNR 3	GCTTGAAGATCTAAAAGTGTTACGCATAACATCTTTTTCTATAC	This study
Amino acid 81–100 deletion in ApiA to construct pSJ104		
DNF 4	GTATAGAAAAAGATGTTATGCGTAACACTGAGTTAGATATTCAG	This study
DNR 4	CTGAATATCTAACTCAGTGTTACGCATAACATCTTTTTCTATAC	This study
Amino acid 101–185 deletion in ApiA to construct pSJ105		
DNF 5	GATTACTAAAAATTTTAGATCTTCAAGCCAAAACATCGCG	This study
DNR 5	CGCGATGTTTTGGCTTGAAGATCTAAAATTTTTAGTAATC	This study
pSJ13 sequence confirmation primers		
pJT1 F	CCT TGC CTA GGG CTA GCA TC	This study
pJT1 R	GGC TGC AGT AAC GAA TAC TAG	This study
*apiA* gene deletion primers		
UF *Not*I	GGGCCCAATTAATGGCCGGTTTGAAATGCACGGTGG	This study
DR *Xho*I	TACTAGTTCGAATAACAGGCGCAG GAATCCGCC	This study
DF	TTAAGGATGAATTTTCACTTAAAGTGCGGTC	This study
UR	GACCGCACTTTAAGTGAAAATTCATCCTTAA	This study
*apiA* gene deletion screening primers		
*api*A^ F	GATATAGCCAGGTGTCTTCGGTGTCG	This study
*api*A^ R	GAATCTTGACCGCGGTGAAGGCATTC	This study
qPCR primers		
*pga*CF	GACGGTGATGCGGTATTGG	This study
*pga*CR	GACCGATGATGGAGCTGAA	This study
*api*AqF	GCCGAGTCAATGAATTAGACAAAG	This study
*api*AqR	CAACAGCTGCACTCAAGTTAAGG	This study
*rcp*AF	TGGGCATTAACTGGAGCCAC	This study
*rcp*AR	ATCCACCTCCGAAACCGAAG	This study
*omp*A1F	GAGATGGCTTGTTGAGAAAC	This study
*omp*A1R	AGGTTATACAGACCGTATCG	This study
*omp*A2 F	CAATATCCGGAGAATAGCGA	This study
*omp*A2 R	GGCATTACGTTTGGAGTATC	This study
*oxy*R F	CTGTAAGGTCGGTACGATATG	This study
*oxy*R R	GCAACCAAGGCAAAGATATG	This study
*kat*A F	GTTCAGCGATCGTGGTATTC	This study
*kat*A R	CGTTGTCGGCATTGATAAAG	This study
5SrRNAF	GCGGGGATCCTGGCGGTGACCTACT	This study
5SrRNAR	GCGATCTAGACCACCTGAAACCATACC	This study

**Table 3:** Auto-aggregation quantified as a measurement of optical density at different time intervals for different strains of *E. coli* expressing variants of *apiA*.

ApiA Construct	0 Min	35 Min	45 Min	60 Min
pET29b+	0.82 ± 04	0.91 ± 03	0.82 ± 04	0.83 ± 05
ApiA WT	0.39 ± 05	0.32 ± 03	0.32 ± 0.1	0.11 ± 12
∆28–33	0.62 ± 08	0.67 ± 14	0.62 ± 0.1	0.52 ± 28
∆34–80	0.62 ± 06	0.60 ± 04	0.52 ± 06	0.51 ± 03
∆81–100	0.45 ± 06	0.14 ± 07	0.16 ± 11	0.19 ± 0.1
∆101–185	0.56 ± 02	0.55 ± 06	0.61 ± 09	0.59 ± 26
∆186–217	0.30+0.23	0.13 ± 08	0.15 ± 07	0.17 ± 05
∆81–185	0.58 ± 08	0.43 ± 36	0.53 ± 24	0.47 ± 18
∆28–185	0.49 ± 07	0.50 ± 09	0.58 ± 04	0.50 ± 09

## Data Availability

Data will be made available upon request.
